# A Central Role for Magnesium Homeostasis during Adaptation to Osmotic Stress

**DOI:** 10.1128/mbio.00092-22

**Published:** 2022-02-15

**Authors:** Brian M. Wendel, Hualiang Pi, Larissa Krüger, Christina Herzberg, Jörg Stülke, John D. Helmann

**Affiliations:** a Department of Microbiology, Cornell University, Ithaca, New York, USA; b Department of General Microbiology, GZMB, Georg August University, Göttingen, Germany; University of Queensland

**Keywords:** osmotic up-shock, osmoadaptation, magnesium homeostasis, c-di-AMP, *Bacillus subtilis*, magnesium, osmotic stress, physiology, potassium transport

## Abstract

Osmotic stress is a significant physical challenge for free-living cells. Cells from all three domains of life maintain viability during osmotic stress by tightly regulating the major cellular osmolyte potassium (K^+^) and by import or synthesis of compatible solutes. It has been widely established that in response to high salt stress, many bacteria transiently accumulate high levels of K^+^, leading to bacteriostasis, with growth resuming only when compatible solutes accumulate and K^+^ levels are restored to biocompatible levels. Using Bacillus subtilis as a model system, we provide evidence that K^+^ fluxes perturb Mg^2+^ homeostasis: import of K^+^ upon osmotic upshift is correlated with Mg^2+^ efflux, and Mg^2+^ reimport is critical for adaptation. The transient growth inhibition resulting from hyperosmotic stress is coincident with loss of Mg^2+^ and a decrease in protein translation. Conversely, the reimport of Mg^2+^ is a limiting factor during resumption of growth. Furthermore, we show the essential signaling dinucleotide cyclic di-AMP fluctuates dynamically in coordination with Mg^2+^ and K^+^ levels, consistent with the proposal that cyclic di-AMP orchestrates the cellular response to osmotic stress.

## INTRODUCTION

It is estimated that half of all enzymes require metals (1), and cells have developed sophisticated mechanisms to regulate the import, intracellular trafficking, and export of metal ions (2). Using Bacillus subtilis as a model system, we have identified the key regulatory proteins that monitor and control the intracellular levels of zinc (Zn^2+^), manganese (Mn^2+^), and iron (Fe^2+/3+^). Ion homeostasis relies on the tight regulation of both import and efflux (2–5), and mutants lacking efflux have an increased sensitivity to metal intoxication (6–10). Previously, we recovered *mpfA* mutations as suppressors of Mn^2+^ sensitivity in strains defective for Mn^2+^ efflux (5, 11). Strains lacking MpfA have an ∼50% increase in intracellular Mg^2+^ (5), consistent with the assignment of MpfA as a major Mg^2+^ efflux system (5, 12). Thus, elevated Mg^2+^ can protect cells against Mn^2+^ intoxication.

In most cells, Mg^2+^ import is tightly regulated. In Escherichia coli, Mg^2+^ import requires the P-type ATPase MgtA. MgtA is under complex regulation, which includes induction through the PhoPQ two-component system and regulation of MgtA activity by the small protein MgtS (13) and allosterically by Mg^2+^ (14). In B. subtilis, Mg^2+^ uptake requires MgtE (15), and *mgtE* expression is controlled at the transcriptional level by a Mg^2+^-sensing riboswitch (16, 17). MgtE activity was allosterically inhibited by Mg^2+^ binding to a cytoplasmic cystathionine beta-synthase (CBS) domain (18), likely in combination with ATP (19). Finally, MgtE stability is tightly regulated by the FtsH intramembrane protease and the YqgP adaptor protein (20).

Since uptake is so tightly regulated, the major physiological role of the MpfA Mg^2+^ efflux protein is not immediately obvious. We hypothesized that Mg^2+^ efflux is elicited by hyperosmotic shock, which often triggers a large influx of K^+^. The level of intracellular Mg^2+^ is second only to that of K^+^, and a large influx of K^+^ (to levels that can approach 1 M) could lead to a displacement of Mg^2+^ from macromolecules and an increase in free Mg^2+^ levels (21). However, most comprehensive reviews of bacterial osmotic stress responses focus on K^+^ influx and make little, if any, mention of how K^+^ might perturb Mg^2+^ pools (22, 23).

Here, we show that hyperosmotic shock triggers a drastic loss of Mg^2+^ from cells. Osmoadaptation is then limited by Mg^2+^ reimport, which in B. subtilis depends on MgtE. In strains deficient in Mg^2+^ export, elevated Mg^2+^ is correlated with a reduction in the basal-level expression of MgtE, and this reduced capacity for Mg^2+^ import contributes to delayed osmoadaptation. We further demonstrate that levels of cyclic di-AMP, a master regulator of osmoadaptation (24), vary, consistent with a role in directly controlling the dynamic and inverse fluctuations of K^+^ and Mg^2+^.

## RESULTS

### Mutants lacking the MpfA Mg^2+^ efflux pump are impaired in osmoadaptation.

**FIG 1:**
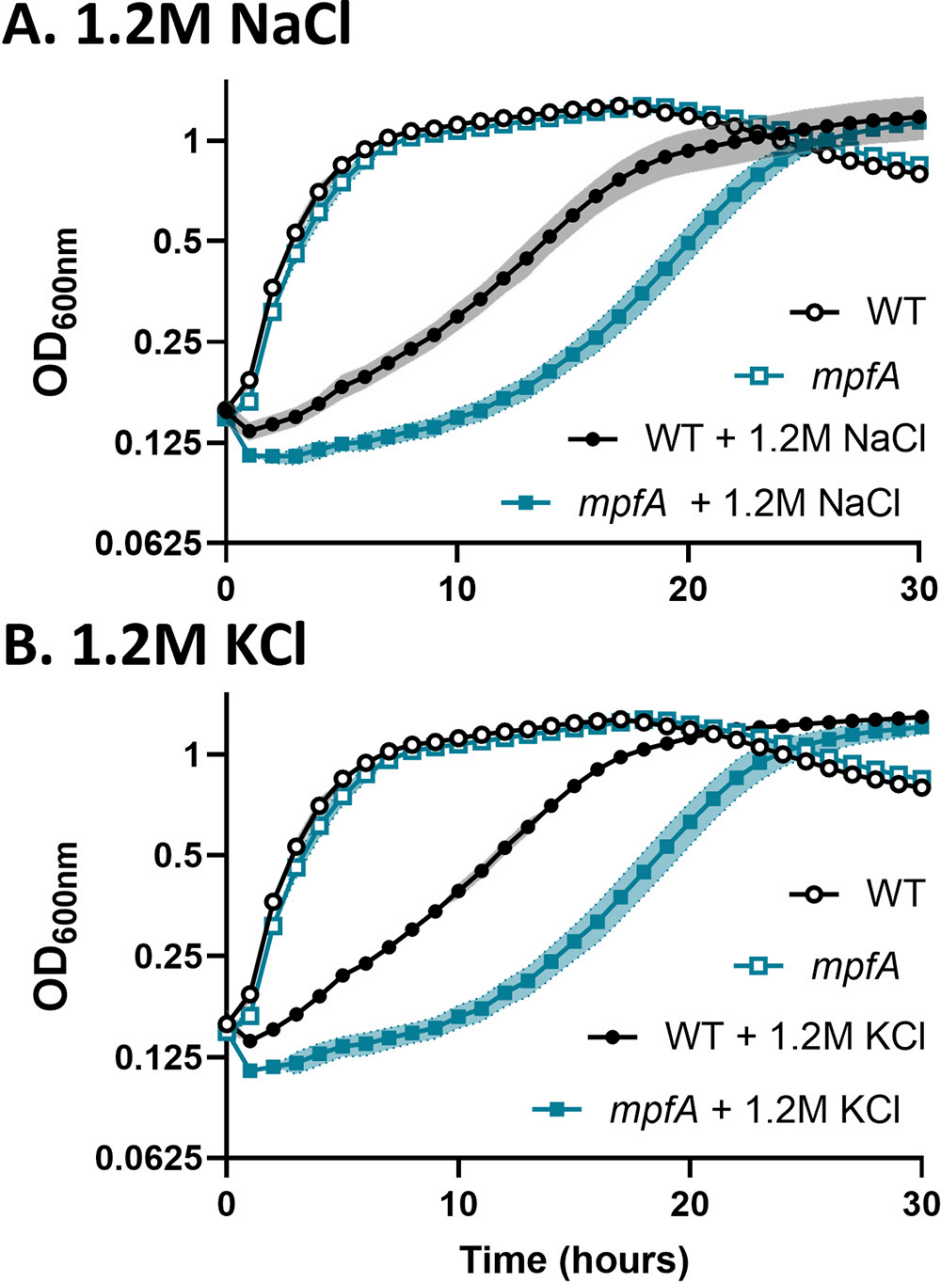
Loss of MpfA impairs osmoadaptation. *mpfA* mutants exhibit a lag in osmoadaptation relative to wild-type cells when exposed to hyperosmotic stress. In minimal medium at 37°C, *mpfA* mutants (blue) grow like WT (black) cells, but they lag in osmoadaptation in medium with 1.2 M NaCl (A) or 1.2 M KCl (B). Exponentially growing cells in minimal medium were subcultured (1:4) in triplicate into the indicated medium. Absorbance at 600 nm was recorded every 60 min. The data shown are the averages (symbols) and standard deviations (shading) from at least three biological replicates. Open symbols represent cells grown on minimal medium, and filled symbols represent cells with the indicated addition.

To study the importance of Mg^2+^ during osmoadaptation, we compared the growth of wild-type B. subtilis strain CU1065 (WT) with an isogenic Δ*mpfA* deletion mutant (here termed *mpfA*) missing the primary Mg^2+^ efflux pump. The subculture of exponentially growing WT cells into a high salt medium elicits osmotic stress and results in a growth lag and reduced growth rate (Fig. 1), as reported previously (25). The *mpfA* strain grew like the WT in the absence of stress but was significantly delayed in osmoadaptation to both high NaCl and high KCl (Fig. 1).

### The role of MpfA in osmoadaptation is correlated with K^+^ import.

**FIG 2:**
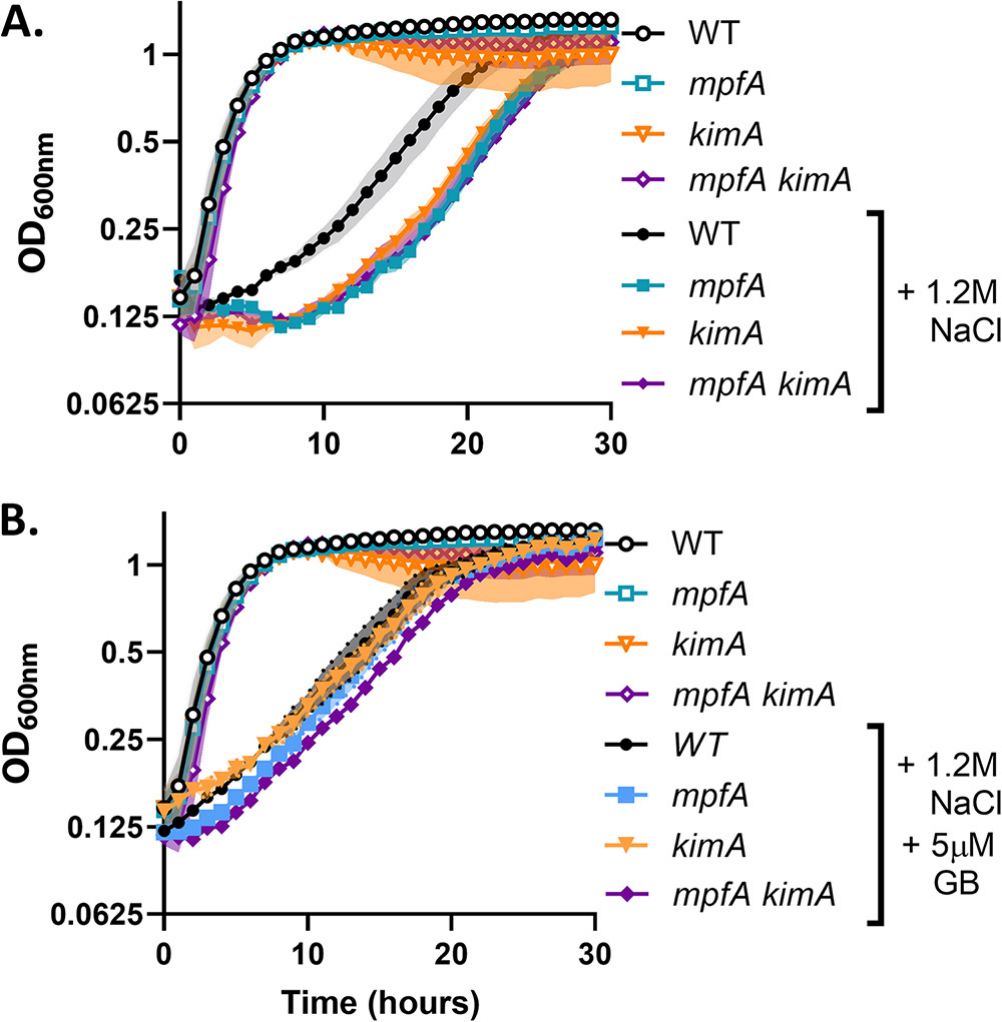
Effects of *kimA* and *mpfA* on osmoadaptation are not additive. (A) Mutants defective in one of the osmotically inducible K^+^ importers, KimA, exhibit a lag in osmoadaptation relative to the WT. This phenotype is not additive with *mpfA*. (B) Addition of 5 μM glycine betaine (GB) reduces the lag in both *mpfA* and *kimA* mutants. This experiment was performed and depicted as indicated in the legend to Fig. 1.

Since osmoadaptation triggers a large influx of K^+^, we hypothesized that MpfA is important for export of displaced Mg^2+^ ions. To test whether the role of MpfA was related to K^+^ import, osmoadaptation was investigated in WT and *mpfA* strains defective in K^+^ import. In agreement with the established importance of K^+^ uptake as a first response to osmotic stress, a mutant lacking the high-affinity K^+^ importer KimA (26) was delayed in osmoadaptation, much like *mpfA*. In the *kimA* strain with reduced K^+^ import, deletion of *mpfA* did not further slow osmoadaptation (Fig. 2A). This is consistent with the reported role of MpfA in Mg^2+^ efflux (5) and the hypothesis that osmotically induced K^+^ import perturbs Mg^2+^ pools.

As reported previously, the compatible solute glycine betaine (GB) modestly increases the rate of osmoadaptation. GB import is known to significantly reduce K^+^ uptake (27). Consistent with reduced K^+^ import, the *kimA* and *mpfA* mutations do not significantly increase the lag time relative to the WT (Fig. 2B). Mutants deficient in the other osmotically inducible K^+^ transporter, KtrAB, exhibited a shorter lag in osmoadaptation, but, like *kimA*, the effect of the *ktrAB* mutation was not additive with *mpfA* (see Fig. S1A in the supplemental material). Notably, *ktrAB kimA* double mutants exhibit slower growth in minimal media without osmotic stress and were unable to adapt and grow in the presence of 1.2 M NaCl, with or without MpfA (Fig. S1B). This supports the idea that KimA and KtrAB are partially redundant in their ability to import K^+^ under osmostress conditions and reinforces the view that this is an essential first response in the absence of compatible solutes (22, 23).

10.1128/mbio.00092-22.1 FIG S1 Osmoadaptation requires osmotically induced K^+^ import. (A) Loss of KtrAB (in strains retaining KimA) has little effect on osmoadaptation in either WT or *mpfA* strains. (B) Mutant strains lacking both high-affinity K^+^ importers (*kimA ktrAB*) have reduced growth rate and fail to adapt to hyperosmotic stress. The experiment was performed and depicted as indicated in the legend to Fig. 1. Download FIG S1, TIF file, 0.1 MB. Copyright © 2022 Wendel et al. 2022 Wendel et al. https://creativecommons.org/licenses/by/4.0/ This content is distributed under the terms of the Creative Commons Attribution 4.0 International license.

### Mg^2+^ levels fluctuate inversely to K^+^ levels during osmoadaptation.

**FIG 3:**
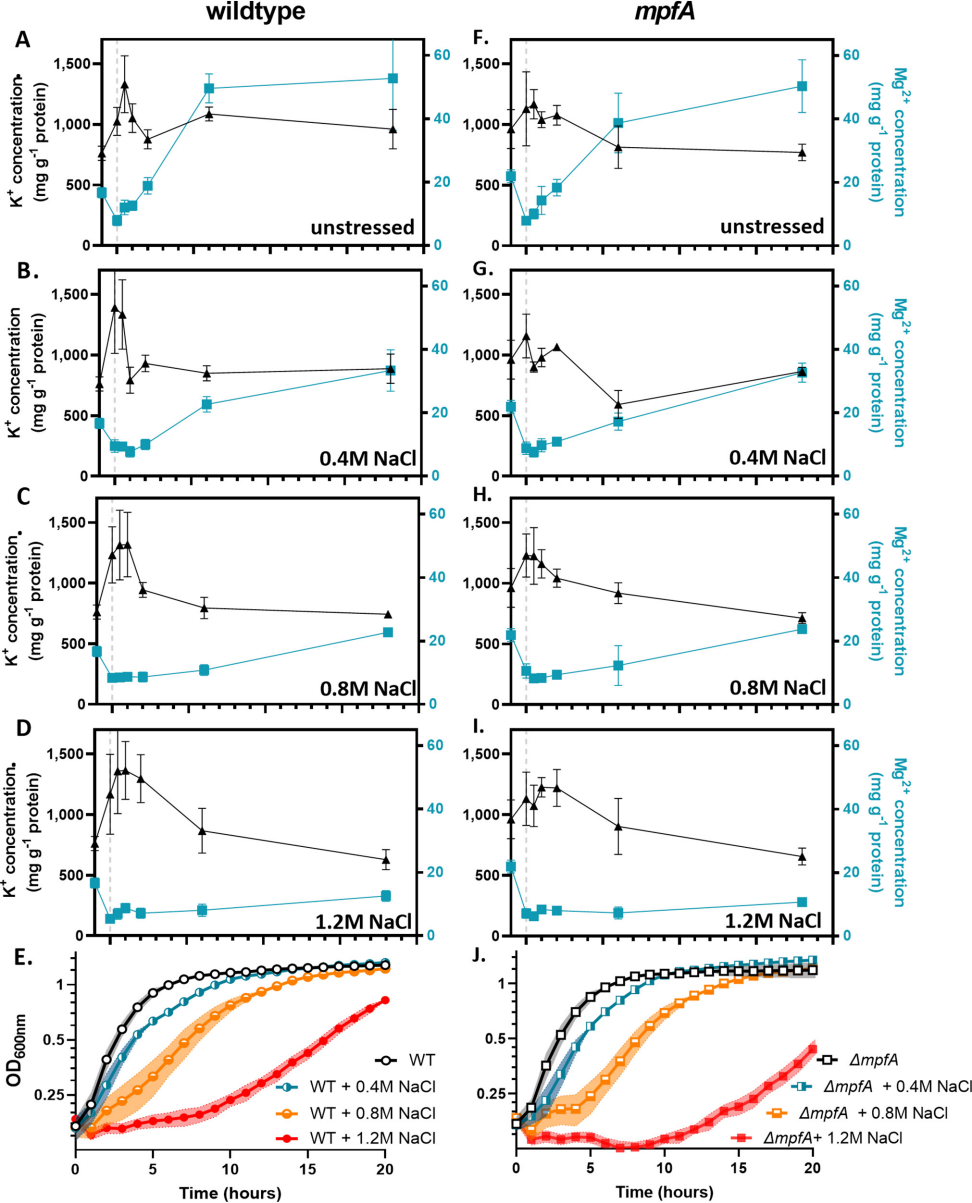
Mg^2+^ and K^+^ levels are inversely correlated during osmoadaptation. Total cellular K^+^ and Mg^2+^ levels were monitored before and after subculture into increasing concentrations of NaCl. (A) WT cells exhibit a rapid spike in K^+^ and simultaneous drop in Mg^2+^, recovering to normal K levels after 2 h. (B to D) As the concentration of NaCl increases, the duration of the fluctuations increase. (E) Growth of WT cells correlates with a restoration of Mg^2+^ levels. (F to I) *ΔmpfA* mutants exhibit patterns similar to those of WT cells. (J) Similar to WT cells, growth of *ΔmpfA* mutants appears to correlate with a restoration of Mg levels. The gray dashed line indicates the point of subculture/addition. Samples for analysis by ICP-MS were taken at the indicated time points and processed as described in Materials and Methods. Growth experiments were performed as indicated in the legend to Fig. 1.

Next, we quantified K^+^ and Mg^2+^ fluxes during osmoadaptation by inductively coupled plasma mass spectrometry (ICP-MS). Consistent with previous literature (21), the K^+^ levels nearly doubled upon exposure to hyperosmotic shock in WT cells (Fig. 3A to D). Surprisingly, even in cells subcultured into fresh medium without added NaCl, K^+^ levels also transiently increased (Fig. 3A), but the duration of this effect was reduced compared to that of the high salt media. Previous studies have also noted that the phenotypic response to nutrient upshift can mimic that of osmotic upshift, consistent with our observation (28). As the level of osmotic stress increased, so did the duration of the K^+^ increase (Fig. 3B to D). This spike in K^+^ was inversely correlated with a precipitous drop in Mg^2+^. In both WT and *mpfA* strains, intracellular Mg^2+^ dropped transiently, but in medium with 1.2 M NaCl this decline was much more persistent. Although we were surprised that the *mpfA* strain still displayed a rapid loss of Mg^2+^, this is likely due to the presence of several paralogs (see Discussion).

We postulate that K^+^ is displacing Mg^2+^, leading to an elevation of free Mg^2+^ pools and triggering efflux. Since ICP-MS measures the total cellular Mg^2+^ content, we turned to the fluorescent probe Mag-Fura-2 to monitor free (readily chelatable) Mg^2+^ in the cytosol. Indeed, free Mg^2+^ levels fall upon osmotic upshift, and this decrease was enhanced when 1.2 M KCl replaced 1.2 M NaCl (Fig. S2). This supports the idea that imported K^+^ is displacing Mg^2+^. We conclude that Mg^2+^ levels fluctuate inversely to K^+^ during osmoadaptation.

10.1128/mbio.00092-22.2 FIG S2 Mg^2+^ is rapidly lost during hyperosmotic shock. Chelatable (free) Mg^2+^ levels were monitored using Mag-Fura2 fluorescence. Loss of intracellular, chelatable Mg^2+^ is greater in cells stressed with 1.2 M KCl than 1.2 M NaCl, consistent with a role for K^+^ import in displacement of bound Mg^2+^ in the cytosol. Data shown are averages and standard deviations from three biological replicates. Download FIG S2, TIF file, 0.1 MB. Copyright © 2022 Wendel et al. 2022 Wendel et al. https://creativecommons.org/licenses/by/4.0/ This content is distributed under the terms of the Creative Commons Attribution 4.0 International license.

### Reimport of Mg^2+^ is critical for adaptation to hyperosmotic stress.

**FIG 4:**
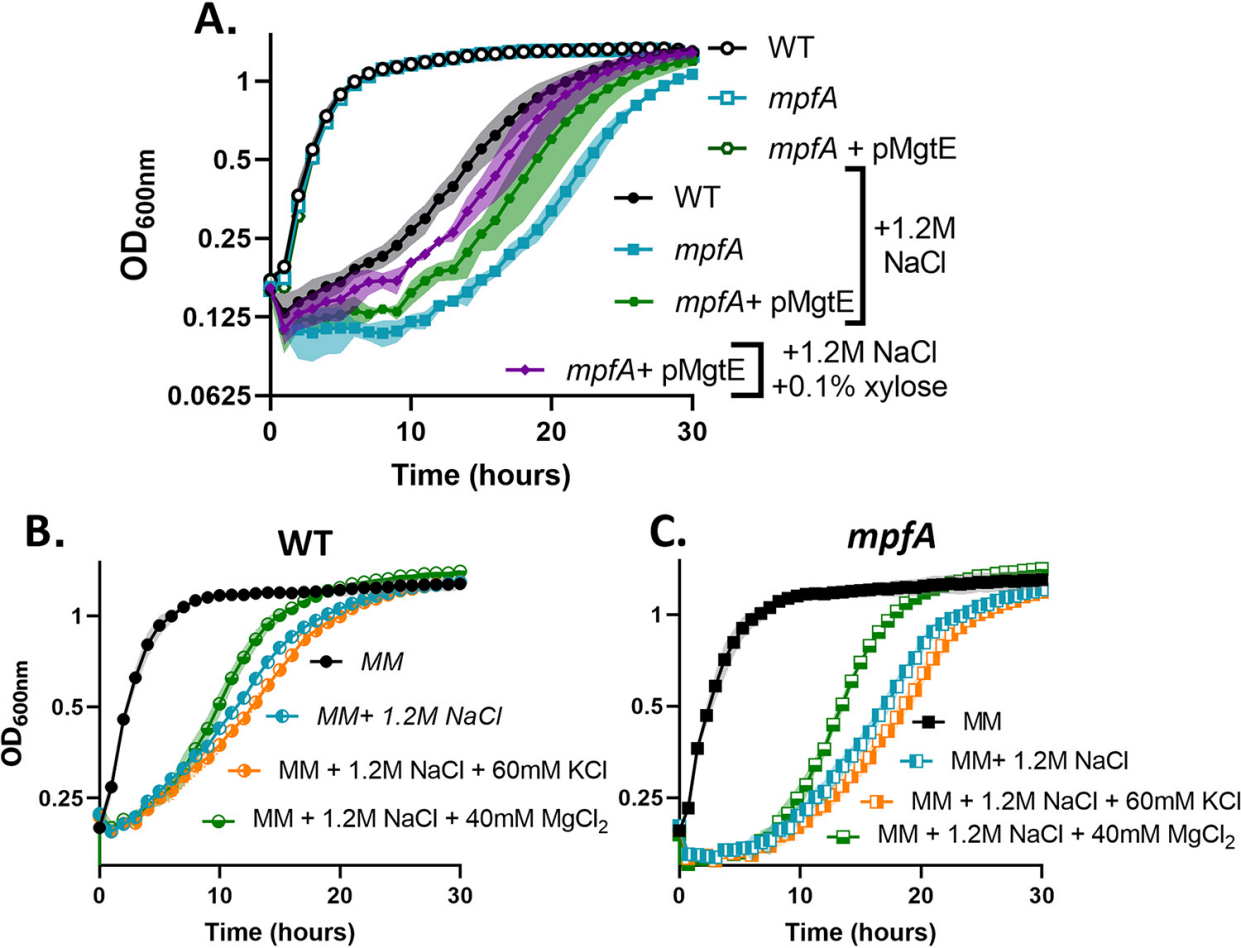
Magnesium reimport is important for osmoadaptation. (A) Expression of MgtE partially rescues the lag in osmoadaptation in Δ*mpfA* mutants. (B and C) Supplementation of 60 mM KCl increases the lag in osmoadaptation in both WT and Δ*mpfA* cells. Supplementation of an equal osmolarity (40 mM) of MgCl_2_ suppresses the lag in osmoadaptation in both WT and Δ*mpfA* cells. Growth experiments were performed and depicted as indicated in the legend to Fig. 1.

We noted that the time required for osmoadaptation (Fig. 3E) seems to be correlated with the time required for the restoration of intracellular Mg^2+^ levels (Fig. 3B to D). This led us to hypothesize that reduced growth under hyperosmotic stress may result from Mg^2+^ limitation rather than a direct effect of high K^+^. To test this idea, we induced MgtE during osmoadaptation using a xylose-inducible promoter. As predicted, increased expression of *mgtE* reduced the lag in osmoadaptation relative to the WT (Fig. 4A). Further, addition of 60 mM KCl (120 mosM) to cells already stressed with 1.2 M NaCl slowed osmoadaptation, whereas addition of 40 mM MgCl_2_ (also 120 mosM) had the opposite effect and actually increased the rate of osmoadaptation (Fig. 4B). A similar effect was observed in *mpfA* mutants (Fig. 4C). In parallel experiments, we tested the ability of an inducible *mpfA* construct to increase the rate of osmoadaptation. In contrast to *mgtE*, induction of *mpfA* at the time of subculture did not increase the rate of osmoadaptation (Fig. S3). Together, these results support Mg^2+^ reimport (and not efflux) as the rate-limiting process during adaptation to hyperosmotic stress.

10.1128/mbio.00092-22.3 FIG S3 Restoration of MpfA does not suppress the delay in osmoadaptation. Expression of MpfA does not rescue the lag in osmoadaptation in Δ*mpfA* mutants. Experiment was performed and depicted as indicated in the legend to Fig. 1. Download FIG S3, TIF file, 0.1 MB. Copyright © 2022 Wendel et al. 2022 Wendel et al. https://creativecommons.org/licenses/by/4.0/ This content is distributed under the terms of the Creative Commons Attribution 4.0 International license.

### Mg^2+^ deficiency during osmoadaptation impairs translation.

**FIG 5:**
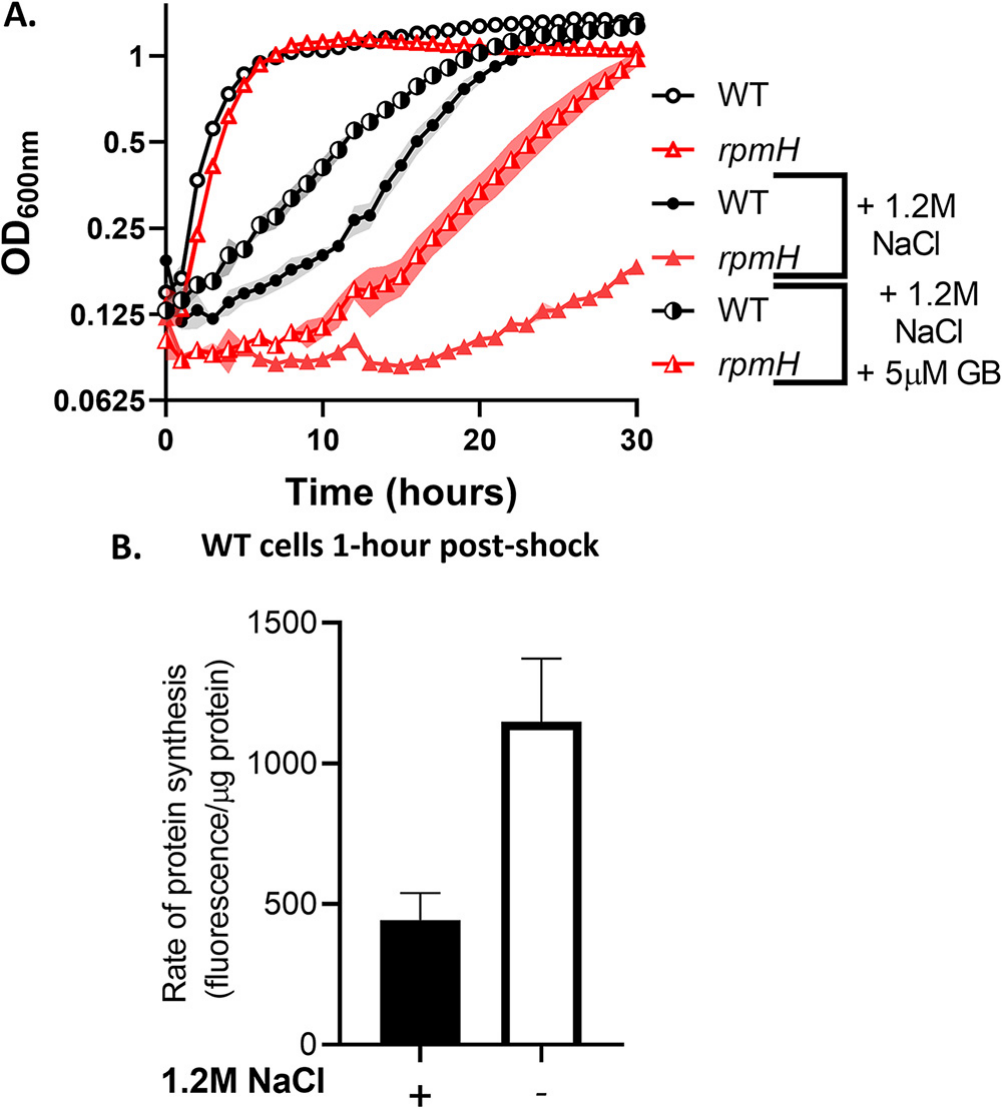
Magnesium limitation during osmoadaptation may contribute to impaired translation. (A) *rpmH* mutants (defective in ribosomal protein L34) exhibit somewhat reduced growth in minimal media and a severe lag relative to the WT under osmotic stress. This growth defect is partially rescued by 5 μM GB. Cells were grown and depicted as indicated in the legend to Fig. 1. (B) The translation rate in the WT was reduced at 1 h after subculture in the presence of 1.2 M NaCl, as measured by l-azidohomoalanine labeling. Data presented are averages and SD from 3 biological replicates (*P* < 0.01).

One likely consequence of Mg^2+^ deficiency is impaired translation (29). Ribosomes contain a significant fraction of the total cellular pool of Mg^2+^ (30, 31), and translation is a major energy-dependent process in the cell fueled by NTP pools, which function as NTP:Mg^2+^ salts (29). Furthermore, *rpmH* mutants deficient in ribosomal assembly due to loss of the large-subunit ribosomal protein L34 are suppressed by supplemental Mg^2+^ or by mutations in *mpfA* that increase cytosolic Mg^2+^ (32). To test whether Mg^2+^ deficiency during osmoadaptation affects translation, we evaluated the response of the *rpmH* mutant to salt stress. The *rpmH* mutant was severely impaired for growth in the presence of 1.2 M NaCl yet was only partially rescued by the addition of the compatible solute glycine betaine (Fig. 5A). Thus, a strain known to have ribosomes sensitive to the depletion of cellular Mg^2+^ pools (32) is strongly affected in osmoadaptation.

To directly evaluate the effect of osmotic upshift on translation, we labeled cells with the methionine analog l-azidohomoalanine after 1 h of subculture into medium with and without 1.2 M NaCl. In the presence of high salt, nascent translation was significantly reduced (Fig. 5B). These data, together with recent publications highlighting the connection between Mg^2+^ homeostasis and translation (29, 33, 34), support the idea that a decrease in Mg^2+^ during osmoadaptation could impair translation and thereby delay the resumption of growth.

### c-di-AMP levels fluctuate dynamically during osmoadaptation.

Cyclic di-AMP has been implicated in growth under osmotic stress due to its central role in coordinating K^+^ homeostasis (26, 35, 36). However, c-di-AMP also binds MgtE (37). To test if c-di-AMP may be regulating Mg^2+^ homeostasis during osmoadaptation, mutants defective in one of the constitutively expressed diadenylate cyclases, CdaA or DisA, or one of the c-di-AMP-specific phosphodiesterases, GdpP or PgpH, were exposed to osmotic upshock. Interestingly, *pgpH* mutants exhibited a lag in osmoadaptation similar to *mpfA* mutants, and the other single mutants also exhibited a lag, but not as much as *mpfA* (Fig. S4A). A *pgpH gdpP* double mutant accumulates toxic levels of c-di-AMP, and this strain rapidly picks up suppressors (38, 39). One of our double mutant strains developed suppressor mutations in *yfkN*, a membrane-bound phosphodiesterase, and *ywfM*, an unknown putative transporter. This *pgpH gdpP* double mutant grew more slowly than the WT and was unable to grow under osmotic stress (Fig. S4B). Interestingly, and consistent with the established inhibition of compatible solute import by c-di-AMP (35, 39, 40), the *pgpH gdpP* double mutant was not rescued by GB (Fig. S4B).

10.1128/mbio.00092-22.4 FIG S4 Perturbations in cyclic-di-AMP metabolism impairs osmoadaptation. (A) Single mutants defective in synthesizing (*cdaA* or *disA*) or degrading (*gdpP* or *pgpH*) c-di-AMP exhibit a lag relative to the WT during osmoadaptation (B). A *pgpH gdpP* double mutant (with high c-di-AMP) is unable to adapt to hyperosmotic shock, even in the presence of GB. This experiment was performed as described in the legend to Fig. 1. Download FIG S4, TIF file, 0.1 MB. Copyright © 2022 Wendel et al. 2022 Wendel et al. https://creativecommons.org/licenses/by/4.0/ This content is distributed under the terms of the Creative Commons Attribution 4.0 International license.

**FIG 6:**
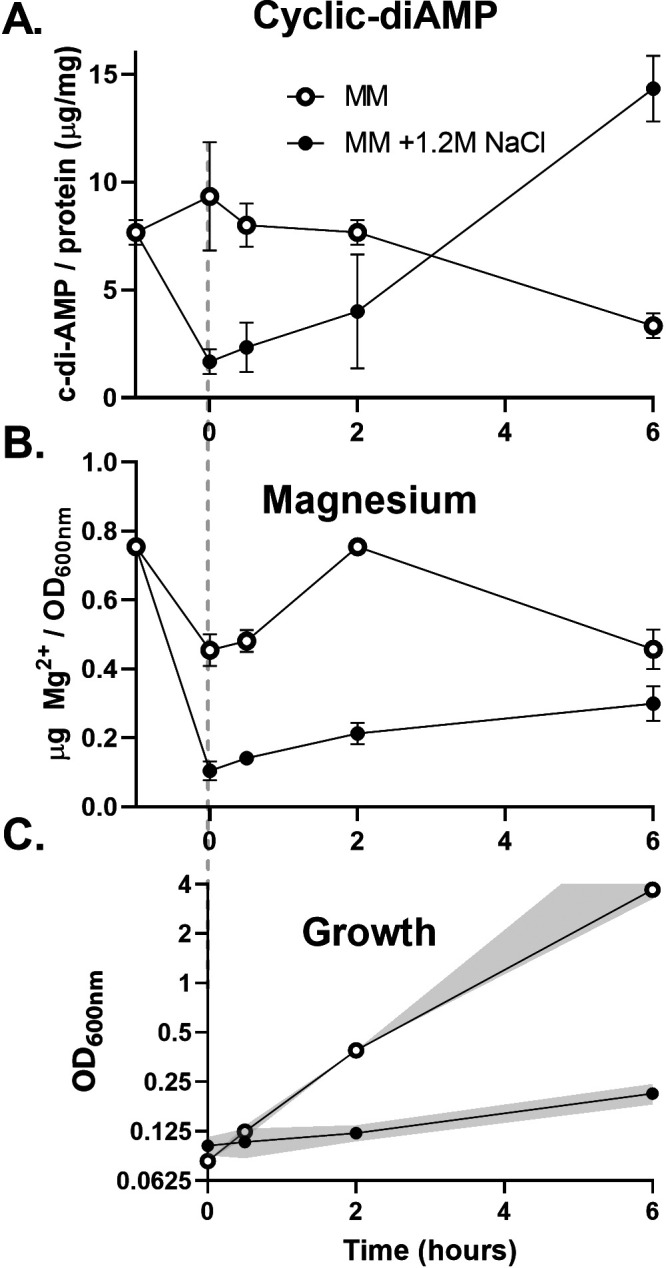
Cyclic-di-AMP levels fluctuate coordinately with Mg^2+^ levels during osmoadaptation. (A) The levels of c-di-AMP levels drop upon subculture with 1.2 M NaCl but not in the absence of hyperosmotic stress. (B) Mg^2+^ levels also drop upon subculture with 1.2 M NaCl. (C) This drop in cell-associated Mg is associated with a reduction in growth rate. The gray dashed line indicates the point of subculture/addition. The plots represent the averages and standard deviations from three biological replicates. The experiment was designed and depicted as indicated in the legend to Fig. 1. Mg levels were measured by ICP-OES and c-di-AMP levels by LC-MS. In this experiment, culture density for cultures with an OD_600_ of >1.0 was determined after 10× dilution and absorbance values calculated accordingly.

Based on the known inhibition of K^+^ and compatible solute import by c-di-AMP (26, 39) and a proposed role in regulating the activity of MgtE (37), we hypothesized that c-di-AMP levels fluctuate dynamically during osmoadaptation. Specifically, an initial decrease in c-di-AMP might facilitate K^+^ and compatible solute import, and a subsequent rise in c-di-AMP may be required for K^+^ efflux, the restoration of Mg^2+^ import, and resumption of growth. Consistent with this, both c-di-AMP and Mg^2+^ levels drop following hyperosmotic shock, and both recover in parallel during osmoadaptation (Fig. 6A to C). Thus, fluctuations in c-di-AMP levels are consistent with a direct role in coordinating K^+^ and Mg^2+^ fluxes during the response to hyperosmotic stress.

## DISCUSSION

**FIG 7:**
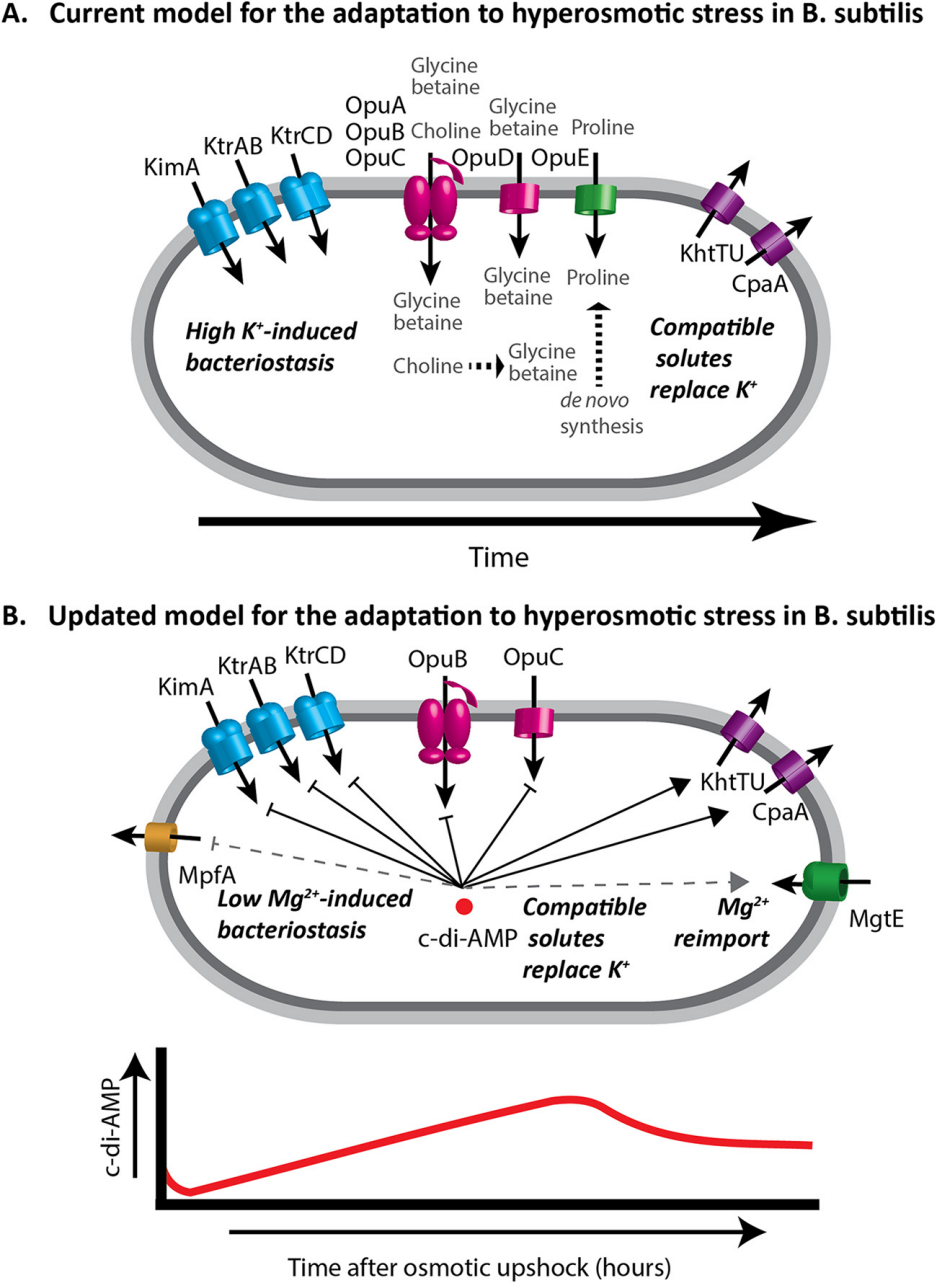
Osmotic stress response in B. subtilis. (A) In the current model, when cells encounter hyperosmotic stress they rapidly import K^+^ to stabilize turgor and retain cellular water. Compatible solutes are then accumulated through import or synthesis and K^+^ is exported. In the presence of compatible solutes K^+^ import is reduced, and turgor is maintained without gross disruption of ion pools. (B) In our updated model, K^+^ import is accompanied by Mg^2+^ loss, and essential cell processes such as translation are thereby inhibited. In the absence of MpfA, Mg^2+^ homeostasis is perturbed and MgtE levels are reduced, which diminishes the capacity for Mg^2+^ reimport, thereby delaying osmoadaptation. (Lower panel) Cyclic-di-AMP varies during osmoadaptation and functions to inhibit expression and activity of K^+^ and compatible solute importers and to activate K^+^ exporters. This signaling nucleotide may also regulate Mg^2+^ homeostasis during osmoadaptation.

Osmotic upshift can restrict cell growth by dehydration of the cytoplasm. In E. coli, the growth rate is linearly correlated with the amount of free water over a wide range of conditions (41). In the current model for osmoadaptation in B. subtilis (Fig. 7A), dehydration is countered by K^+^ uptake, mediated by KimA and KtrAB, which can raise the intracellular K^+^ concentration to levels approaching 1 M (21, 26, 42, 43). This import is transient, as high K^+^ is proposed to compromise protein function and membrane potential (44). To rebalance the ionic strength of the cytoplasm (45), B. subtilis imports compatible solutes such as glycine betaine (GB) and proline (46, 47). If extracellular osmolyte concentrations are insufficient, then cells defer to the energetically costly *de novo* synthesis of proline (48). Finally, K^+^ efflux is proposed to facilitate the resumption of essential cytoplasmic functions and cell growth (49, 50). Here, we amend this model by integration of Mg^2+^ and c-di-AMP as central players in osmoadaptation (Fig. 7B).

Mg^2+^ has not been previously implicated as a major player during osmoadaptation, in part because its concentration (unlike K^+^) is too low to have a significant role as an osmolyte (44). Indeed, Mg^2+^ homeostasis is rarely mentioned in discussions of bacterial osmoadaptation (22, 51). However, previous work suggests that osmotic stress and the accompanying rise in K^+^ levels can perturb intracellular ion pools. For example, in E. coli osmotic stress triggers proton egress and a rise in intracellular pH in E. coli (52), and in osmotically stressed human (HeLa) cells a transient rise in Mg^2+^ levels was noted (53). A coupling between osmotic stress and Mg^2+^ pools has also been suggested from system-level modeling of the bacterial metabolome (54).

We hypothesized import of K^+^ upon osmotic upshift would displace Mg^2+^ from complexes within the cell, and that this displaced Mg^2+^ would be lost from the cell through efflux. In support of this idea, *mpfA* mutants are delayed in osmoadaptation, and this delay appears to be related to K^+^ influx (Fig. 1 and 2). We further anticipated that *mpfA* mutant cells would be defective in Mg^2+^ efflux and perhaps impaired in K^+^ import. However, that is clearly not the case (Fig. 3), and slower osmoadaptation in the *mpfA* strain is not correlated with an obvious defect in Mg^2+^ efflux. Although at first puzzling, we realized that a key difference between the WT and *mpfA* cells might instead be the rate of Mg^2+^ reimport (i.e., Fig. 3B versus G). We noted that prior to osmotic upshift, *mpfA* cells have ∼36% increased intracellular Mg^2+^ levels (see Table S3 in the supplemental material), consistent with the ∼50% increase noted previously (5). Since MgtE is rate-limiting for the resumption of growth (Fig. 4A) and *mgtE* transcription is regulated by a Mg^2+^-sensing riboswitch (16), we hypothesize that *mpfA* mutants have a reduced capacity for Mg^2+^ uptake. Indeed, *mgtE* mRNA levels, monitored by reverse transcription-PCR (RT-PCR), were reduced >2-fold in the *mpfA* mutant (Fig. S5). This striking decrease, despite a more modest change in Mg^2+^ levels, might result from cooperativity of Mg^2+^ binding to the MgtE riboswitch, as proposed previously (55, 56). The persistence of Mg^2+^ efflux in the *mpfA* strain is likely due to alternative efflux systems. Indeed, B. subtilis encodes four MpfA paralogs (YrkA, YhdT, YqhB, and YugS), and mutations in any one of these paralogs also lead to a lag in osmoadaptation, similar to the *mpfA* strain (Fig. S6A), and in each case the observed lag was suppressed by the addition of GB (Fig. S6B), which is known to reduce K^+^ import (21). We conclude that mutations affecting known (MpfA) or candidate Mg^2+^ efflux proteins all delay osmoadaptation and that these proteins are partially redundant with respect to Mg^2+^ egress. Delayed osmoadaptation in these strains highlights the importance of Mg^2+^ homeostasis and argues for an amendment to our current understanding of bacterial osmostress responses (Fig. 7B).

10.1128/mbio.00092-22.5 FIG S5 *ΔmpfA* mutants have reduced expression of the MgtE Mg^2+^ importer. MgtE expression is reduced in *ΔmpfA* mutants relative to the WT as measured using RT-PCR. Gene expression values (2^−Δ^*^CT^*) of *mgtE* were normalized to *gyrA* for WT and *ΔmpfA* strains (*P* < 0.01). Download FIG S5, TIF file, 0.2 MB. Copyright © 2022 Wendel et al. 2022 Wendel et al. https://creativecommons.org/licenses/by/4.0/ This content is distributed under the terms of the Creative Commons Attribution 4.0 International license.

10.1128/mbio.00092-22.6 FIG S6 Loss of MpfA paralogs delays osmoadaptation. (A) Single mutants lacking any one MpfA paralog demonstrate a lag in osmoadaptation. (B) The growth lag in the mutant strains is suppressed by the presence of a compatible solute (glycine betaine, or GB). Plots are the average (line/point) and SD (shading) from three biological replicates. Open symbols indicate minimal medium with no additives, and solid symbols indicate minimal medium with 1.2 M NaCl or 1.2 M NaCl and 5 μM GB. Download FIG S6, TIF file, 2.1 MB. Copyright © 2022 Wendel et al. 2022 Wendel et al. https://creativecommons.org/licenses/by/4.0/ This content is distributed under the terms of the Creative Commons Attribution 4.0 International license.

10.1128/mbio.00092-22.9 TABLE S3 Intracellular metal content of exponentially growing cells. Download Table S3, DOCX file, 0.01 MB. Copyright © 2022 Wendel et al. 2022 Wendel et al. https://creativecommons.org/licenses/by/4.0/ This content is distributed under the terms of the Creative Commons Attribution 4.0 International license.

Mg^2+^ homeostasis is tightly regulated by both uptake and export. B. subtilis MgtE is an essential transporter required for regulated, high-affinity import (15). Expression of *mgtE* is transcriptionally regulated by a Mg^2+^-sensitive riboswitch, and MgtE activity is feedback inhibited by Mg^2+^ (16, 18, 57, 58). Despite the long history of work on Mg^2+^ homeostasis (59), Mg^2+^ efflux pumps were only recently identified (5, 12). MpfA was discovered in genetic screens for suppressors of ribosome assembly defects and metal intoxication (5, 33, 60, 61). A Listeria monocytogenes homolog, *lmo233*, was reported to be important for growth in high salt prior to recognition of its role in Mg^2+^ homeostasis (62). MpfA is now appreciated as a Mg^2+^ efflux pump in both B. subtilis and S. aureus (5, 63). Interestingly, B. subtilis encodes four MpfA paralogs (5), all with salt-responsive transcriptional regulation (64).

Given the central role of Mg^2+^ in cell physiology and the large-scale perturbation of the cellular metallome by K^+^ import during hyperosmotic stress, we monitored changes in Mg^2+^ during a time course of osmoadaptation. We demonstrate that osmotically induced changes in K^+^ and Mg^2+^ levels are inversely correlated. The depletion of free Mg^2+^ early during osmoadaptation is expected to impair energy-requiring processes in the cell by virtue of the role of free Mg^2+^ as a cofactor for NTPs. Translation is the single most energy-intensive process in the cell, and Mg^2+^-limited cells may become growth-limited due to defects in translation (29). Indeed, an *rpmH* strain in which ribosomes have an elevated requirement for Mg^2+^ is impaired in osmoadaptation (Fig. 5A). We further show that translation is reduced after osmotic upshift (Fig. 5B). Thus, osmotic upshift reduces translation, and a mutation that renders translation more sensitive to a reduction in Mg^2+^ levels slows osmoadaptation. Conversely, conditions that increase reimport of Mg^2+^ increase the rate of osmoadaptation (excess Mg^2+^, overexpression of MgtE). These results support a model in which hyperosmotic stress triggers Mg^2+^ depletion as the proximate cause of bacteriostasis, and Mg^2+^uptake is then limiting for recovery (Fig. 7B).

Recently, the dinucleotide second messenger c-di-AMP has been implicated in the control of K^+^ and compatible solute transport, suggesting that it acts as a central regulator of osmoadaptation (24). Increased c-di-AMP levels inhibit both transcription and activity of the osmotically induced K^+^ transporters KimA and KtrAB and activate the K^+^ exporters CpaA and KhtU (36, 37, 65). Furthermore, increased c-di-AMP inhibits compatible solute uptake by the Opu-family proteins (35, 46). c-di-AMP often binds to proteins that have a CBS (cystathionine-beta-synthase) or an RCK_C (regulator of conductance of K^+^) domain (24). MgtE contains a CBS domain, as do MpfA and its paralogs. c-di-AMP binds to MgtE (37), which supports a role in controlling both Mg^2+^ as well as K^+^ homeostasis. Indeed, upon osmotic upshift c-di-AMP levels are rapidly reduced (Fig. 6A), which, based on our current understanding of c-di-AMP regulation, would facilitate K^+^ and/or compatible solute import (24, 65). During osmoadaptation, c-di-AMP levels rise again, which would reduce K^+^ and compatible solute import, activate K^+^ efflux by KhtTU and CpaA (27, 48), and may serve to activate MgtE-dependent Mg^2+^ reimport, which then allows a resumption of growth (37). Whether or not c-di-AMP also regulates the activity of MpfA and its paralogs remains to be determined (Fig. 7B). Thus, c-di-AMP likely choreographs these responses by regulating both transcription and activity of the transporters for K^+^, Mg^2+^, and compatible solutes throughout osmoadaptation (Fig. 7B).

## MATERIALS AND METHODS

### Bacterial strains and growth conditions.

All strains used in the study are derivatives of B. subtilis strain CU1065 (WT), are listed in Table S1 in the supplemental material, and were verified using primers listed in Table S1. Gene replacement cassettes were obtained through the Bacillus Genetic Stock Center from the BKE collection (66). Cells were grown in liquid LB, on solid LB agar plates with appropriate antibiotic selection, or in minimal media adapted by Chen et al. from Belitsky minimal medium with vigorous shaking at 37°C (67, 68). Briefly, the minimal media consisted of 15 mM (NH_4_)_2_SO_4_, 1.6 mM MgSO_4_, 4.5 mM potassium glutamate, 40 mM morpholinepropanesulfonic acid (MOPS), pH 7.4, 5 mM KPO_4_, pH 7, 49 mM tryptophan, 2% glucose. The antibiotics (concentrations) used are the following: ampicillin (amp; 100 μg mL^−1^), chloramphenicol (cm; 10 μg mL^−1^), kanamycin (kan; 15 μg mL^−1^), neomycin (neo; 8 μg mL^−1^), and macrolide lincosamide-streptogramin B (MLS; 1 μg mL^−1^ erythromycin and 25 μg mL^−1^ lincomycin). For the minimal media used for c-di-AMP null strains, the MgSO_4_ concentration was raised to 20 mM, the potassium glutamate was omitted, and KH_2_PO_4_ was replaced with NaH_2_PO_4_, as in reference 37.

10.1128/mbio.00092-22.7 TABLE S1 Strains and plasmids used in this study. Download Table S1, DOCX file, 0.02 MB. Copyright © 2022 Wendel et al. 2022 Wendel et al. https://creativecommons.org/licenses/by/4.0/ This content is distributed under the terms of the Creative Commons Attribution 4.0 International license.

### Growth curves.

Cells were grown overnight in LB medium, subcultured at a 1:100 ratio into minimal medium, and grown to exponential phase (optical density at 600 nm [OD_600_], ∼0.4). Cells were subsequently subcultured 1:4 into a prewarmed Bioscreen plate with the indicated conditions. Cell growth (OD_600_) was monitored every 15 min for 30 h using a Bioscreen growth analyzer (Growth Curves USA, Piscataway, NJ) at 37°C with continuous shaking. In Fig. 6, cultures were measured by hand in a spectrophotometer, with densities at an OD_600_ of >1 diluted and values calculated accordingly. Data shown are averages and standard deviations or representative plots from at least three biological replicates.

### RNA extraction and qPCR.

Gene expression for *mgtE* was determined by real-time PCR using primers mentioned in Table S2. RNA was purified from 1.5 mL of exponentially growing cells (OD_600_ of ∼0.4) in minimal media using an RNeasy kit from Qiagen per the manufacturer’s instructions. Two micrograms of RNA was used to prepare 20 μL of cDNA to achieve a final concentration of 100 ng/μL using a high-capacity cDNA reverse transcription kit from Applied Biosystems. The gene expression levels were measured using 100 ng of cDNA using 0.5 μM gene-specific primers and 1× SYBR green (Bio-Rad) in a Quantstudio 7 Pro. Gene expression values (2^−Δ^*^CT^*) were plotted after normalization with *gyrA*. A Student’s *t* test was performed to determine statistical significance.

10.1128/mbio.00092-22.8 TABLE S2 Primer oligonucleotides. Download Table S2, DOCX file, 0.01 MB. Copyright © 2022 Wendel et al. 2022 Wendel et al. https://creativecommons.org/licenses/by/4.0/ This content is distributed under the terms of the Creative Commons Attribution 4.0 International license.

### Quantification of intracellular metal content by ICP-MS.

Cells were grown in LB medium overnight and subcultured at a 1:100 ratio into fresh minimal medium to an OD_600_ of ∼0.4. Cells were then subcultured 1:4 into fresh prewarmed minimal medium with or without the indicated osmotic stressor. Cells were harvested at the indicated time points, and levels of intracellular metals (K, Mg, Fe, Mn, Zn, and Co) were monitored at each time point by inductively coupled plasma mass spectrometry (ICP-MS). All samples were washed once with Chelex-treated phosphate-buffered saline (PBS) buffer. Cell pellets were resuspended in 400 μl of buffer 2 (1× Chelex-treated PBS buffer, 75 mM NaN_3_, 1% Triton X-100) and incubated at 37°C for 90 min to lyse the cells. Lysed samples were spun down by centrifugation, and the total protein content was quantified using a Bradford assay. The samples then were mixed with 600 μl buffer 4 (5% HNO_3_, 0.1% [vol/vol] Triton X-100) and heated in a 95°C sand bath for 30 min. The debris was removed by centrifugation, and the total metal ions in the diluted samples were analyzed by a Perkin-Elmer Elan DRC II ICP-MS. Gallium was used as an internal standard. The total cellular ion levels are expressed as total molar content (means ± standard errors; *n* = 3). An average cell volume of 0.9 μm^3^ and average cell protein content of 0.121 pg was used to determine molarity from the ICP-MS unit in micrograms per gram of protein (69, 70).

### Quantification of intracellular metal content by ICP-OES.

B. subtilis cells were harvested by centrifugation (3 min, 4°C, 8,500 × *g*). Cell pellets were washed twice with Na-PBS buffer and transferred onto ash-free filter discs (pore size, 0.45 mm; diameter, 47 mm). The cells were dried overnight at room temperature, followed by 3 h at 70°C. The dried filter discs were cut into small pieces and reduced into a fluid state through pressure and 2 mL of 65% HNO_3_ for 7 h at 185°C in Teflon beakers (25 mL) (PDS-6 pressure digestion system; Loftfield). After the digestion process, the fluid content in the beakers was transferred into an Erlenmeyer flask and diluted with demineralized water to a volume of 50 mL. The total potassium and magnesium content of the bacterial cells in this solution was determined by ICP-OES analysis (Optima 5300 DV; PerkinElmer). This common type of emission spectroscopy technique uses the ICP to produce element atoms and ions that emit electromagnetic radiation at wavelengths of specific characteristics of a particular chemical element. The intensity of light emission at 766.49 nm and 285.21 nm indicates the potassium and magnesium concentration, respectively. The plasma is built by argon gas ionized in an intense electromagnetic field at a temperature of about 7,000 to 10,000°C, generated as the result of the collisions between the neutral argon atoms and the charged particles (71).

### Quantification of free magnesium by Mag-Fura 2.

Cells were treated with modifications as described in reference 72. Briefly, overnight cultures of cells were diluted into minimal media to an OD_600_ of 0.2 in the presence of the acetoxymethyl ester form of Mag-Fura 2. Cells were loaded with AM-Mag-Fura-2 at a final concentration of 5 μM with 15 μM pluronic F-127 as a cell permeant. After a 75-min loading incubation at 37°C with shaking, cells were washed 2× with prewarmed minimal medium, and 100 μL of cell suspension was added to a 96-well plate. After a 30-min incubation at 37°C with shaking, fluorescent signals in samples were measured in a Synergy H1 reader (BioTek) at 37°C for the bound (340-nm excitation and 509-nm emission) and unbound (380-nm excitation and 509-nm emission) form of Mag-Fura 2 at the minimum interval. Additives dissolved in 1× minimal medium were used as indicated. The ratio of bound to unbound fluorescence signal was plotted.

### AHA labeling of nascent proteins.

Strains were grown overnight in rich medium. The following day, cells were subcultured (1:100) into fresh MM to an OD_600_ of 0.4. Cells were again subcultured (1:4) in MM with and without 1.2 M NaCl. At the indicated time points, cultures were labeled with 400 μM l-azidohomoalanine (AHA) (Click Chemistry Tools) for 30 min. Cultures were treated with 100 μg/mL^−1^ chloramphenicol at the end of the labeling and collected by centrifugation at 4°C. Cells were washed 3× with ice-cold PBS and stored at −80°C. Cell pellets were thawed and resuspended in a lysis buffer consisting of 1 mg/mL^−1^ lysozyme, 50 mM Tris-HCl, pH 8.0, 0.5% SDS. Cells were lysed by sonication, and insoluble debris was removed by centrifugation (10 min, 10,000× rpm, 4°C). Covalent attachment of fluorescent tetramethylrhodamine (TAMRA)-alkyne (Thermo Fisher Scientific) to AHA-containing proteins was carried out using a Click-iT protein reaction buffer kit (Thermo Fisher Scientific) according to the manufacturer's instructions. Protein concentrations were determined by Bradford assay. Fluorescent signals in samples were measured in a Synergy H1 reader (BioTek, VT) with 545-nm excitation and 580-nm emission wavelengths. The fluorescence signal was normalized by the protein content of the sample to determine the translation rate. A Student’s *t* test was performed to determine statistical significance.

### Analysis of cyclic-di-AMP pools.

The concentration of c-di-AMP in B. subtilis cells was determined by a liquid chromatography–tandem mass spectrometry method, as described previously (38). The cells were harvested by centrifugation (4°C, 8,500 × g), shock-frozen in liquid nitrogen, and stored at −80°C. This sample was used for c-di-AMP extraction (38). The chromatographic separation was performed on a Series 200 HPLC (high-performance liquid chromatography) system (PerkinElmer Life Sciences) or an LC-10AD HPLC system (Shimadzu), as described previously (73). Detection of c-di-AMP was performed on an API 3000 or API 4000 triple quadrupole mass spectrometer equipped with an electrospray ionization source (AB Sciex) using selected reaction monitoring (SRM) analysis in positive ionization mode. The SRM transitions labeled as “quantifier” were used to quantify the compound of interest, whereas “identifier” SRM transitions were monitored as confirmatory signals. The quantifier SRM transitions were most intense and used for quantification.
