# Supplementary figures and images for: A Central Role for Magnesium Homeostasis during Adaptation to Osmotic Stress
Source: mBio. 2022 Feb 15;13(1):e00092-22. doi: 10.1128/mbio.00092-22 (PMC8844918; doi:10.1128/mbio.00092-22)

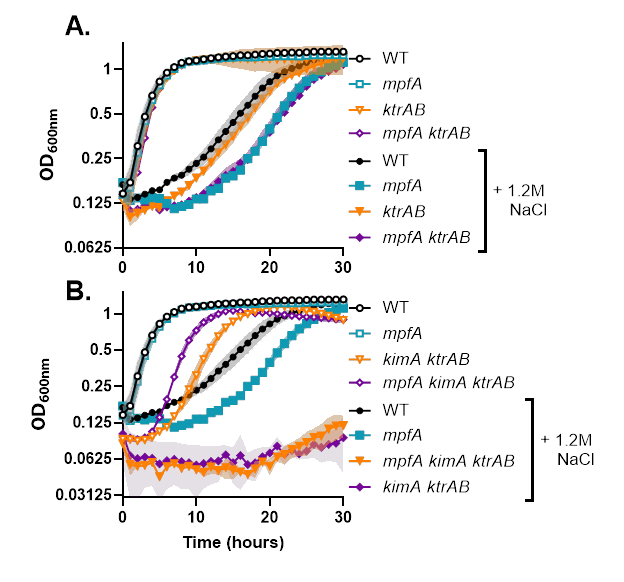

Supplement: FIG S1 [file mbio.00092-22-sf001.tif]

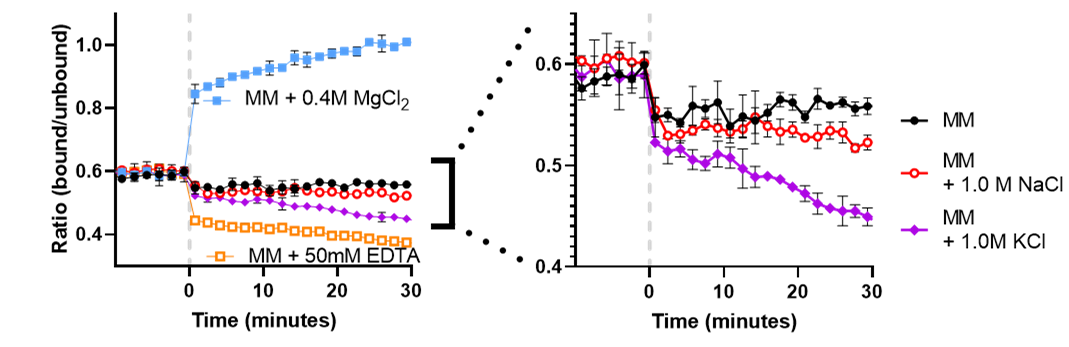

Supplement: FIG S2 [file mbio.00092-22-sf002.tif]

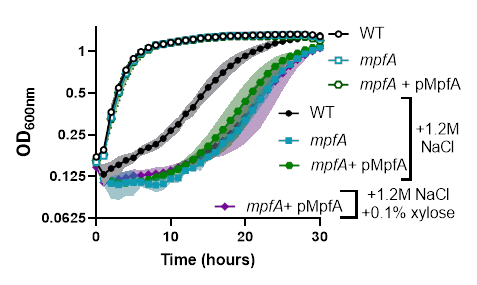

Supplement: FIG S3 [file mbio.00092-22-sf003.tif]

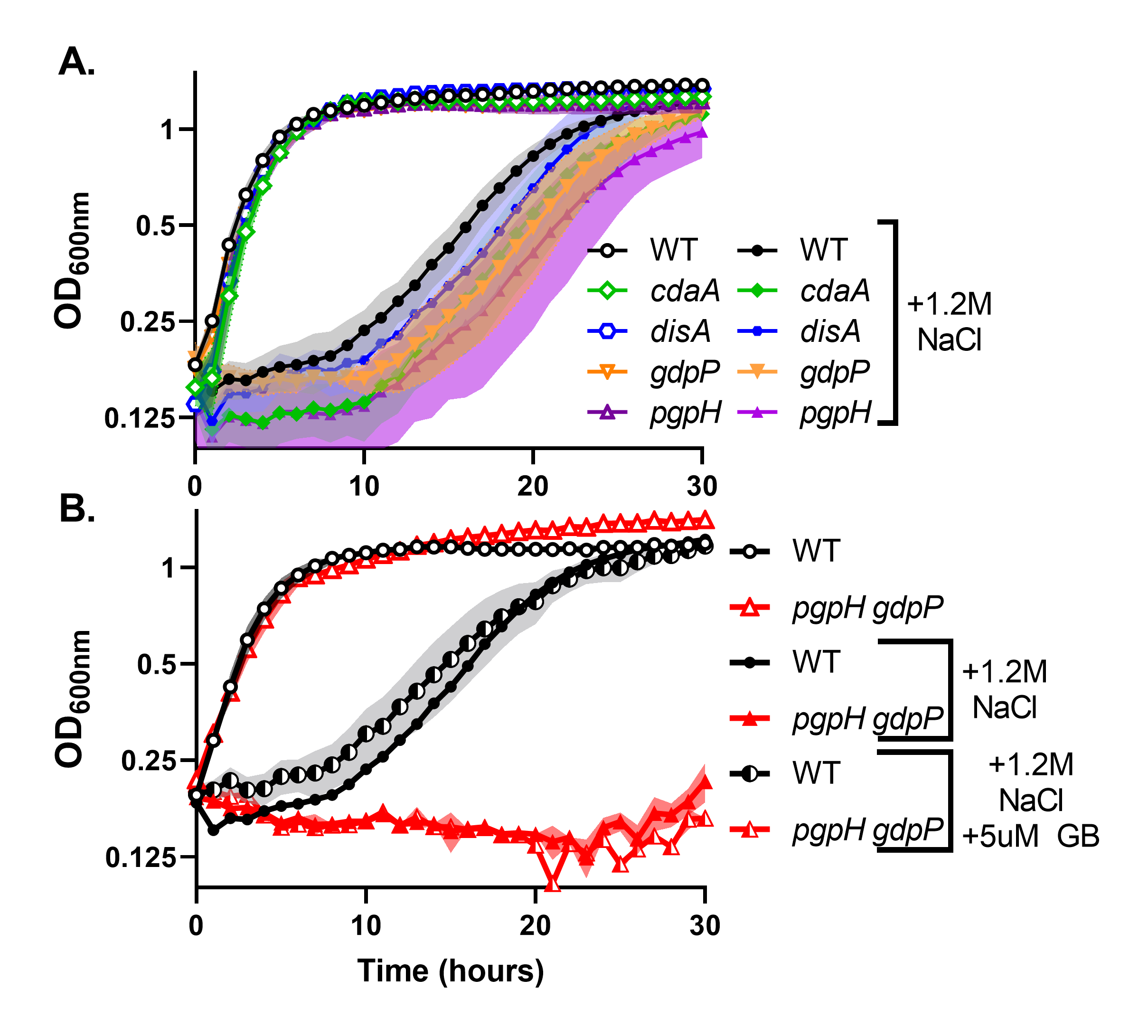

Supplement: FIG S4 [file mbio.00092-22-sf004.tif]

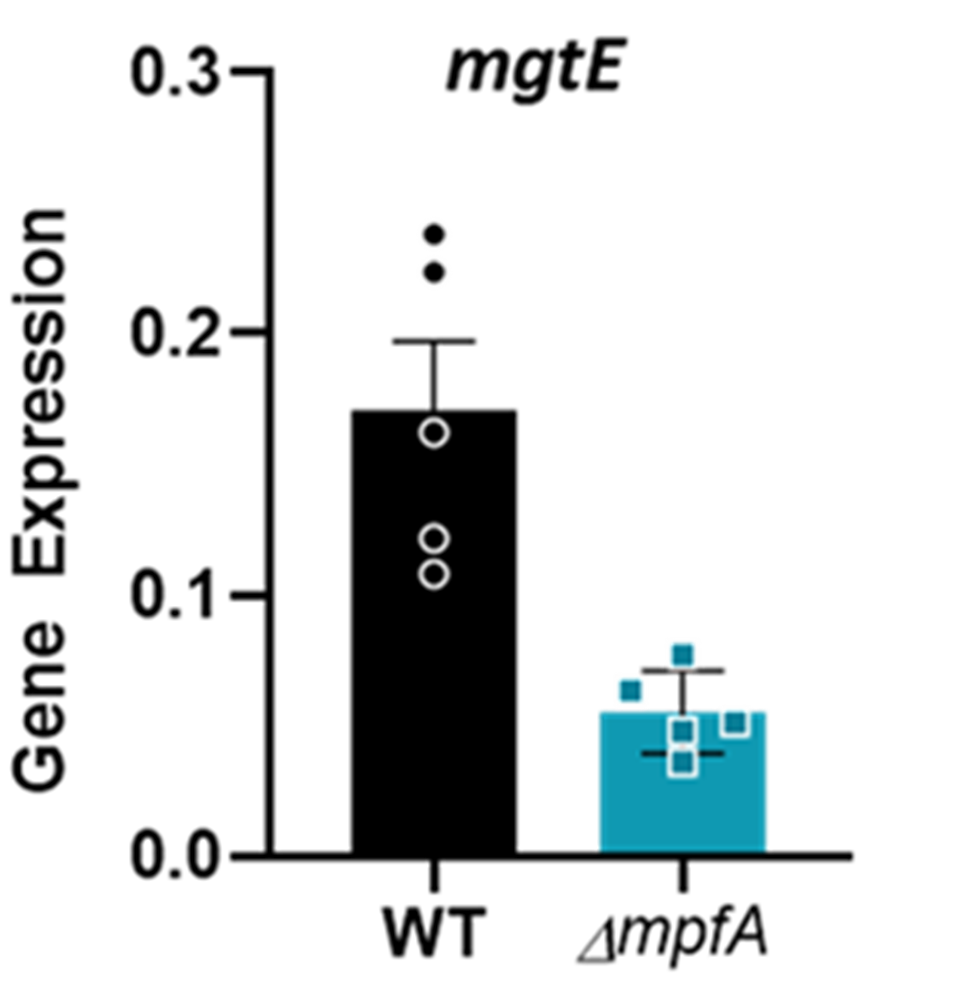

Supplement: FIG S5 [file mbio.00092-22-sf005.tif]

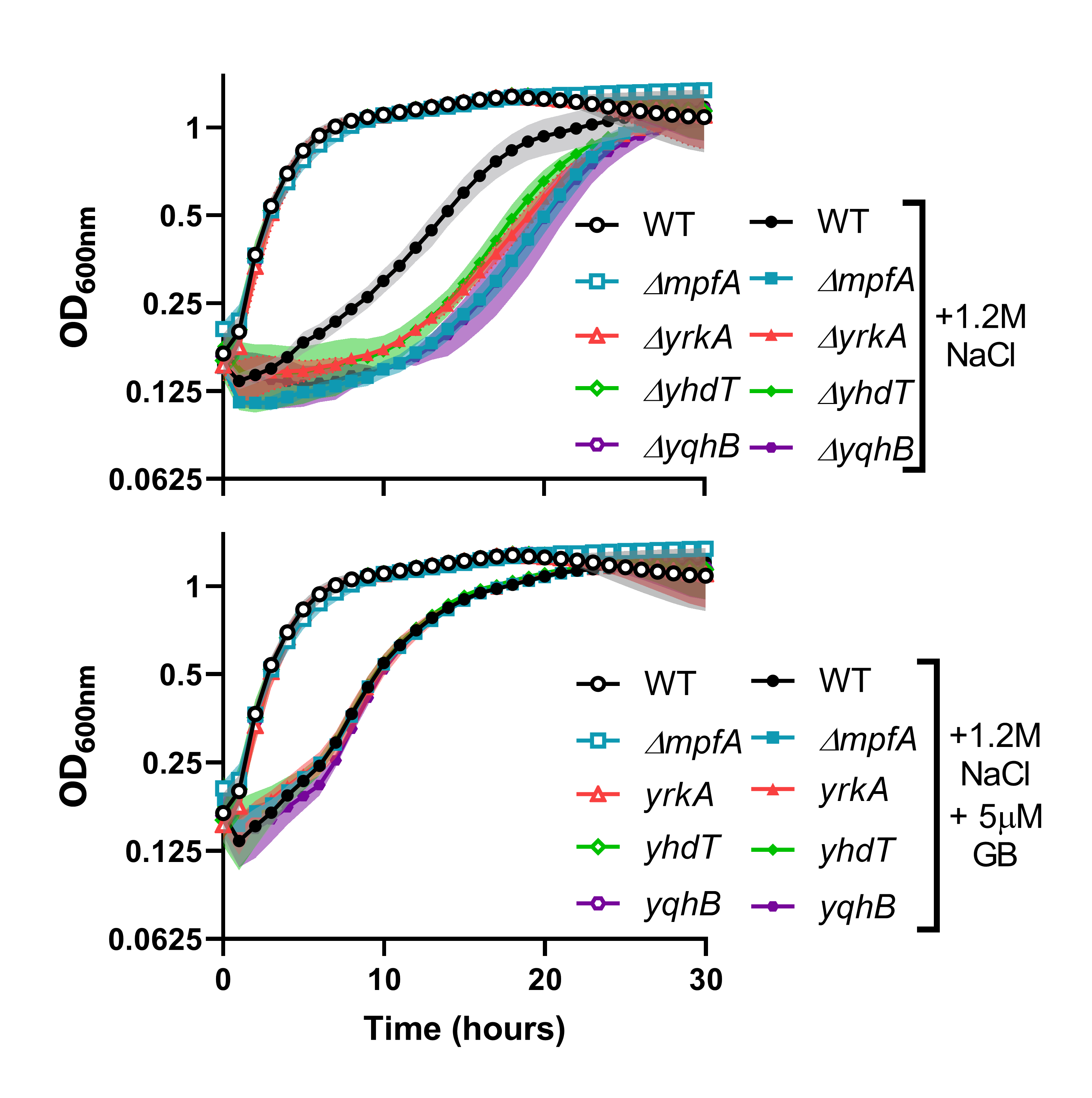

Supplement: FIG S6 [file mbio.00092-22-sf006.tif]
