# Supplementary material for: A Central Role for Magnesium Homeostasis during Adaptation to Osmotic Stress
Source: mBio. 2022 Feb 15;13(1):e00092-22. doi: 10.1128/mbio.00092-22 (PMC8844918; doi:10.1128/mbio.00092-22)
Supplement: TABLE S3 [file mbio.00092-22-st003.docx]

Table S3.

Intracellular metal content of exponentially growing cells.

|  | **Mg** | **K** | **Fe** | **Mn** | **Zn** |
| --- | --- | --- | --- | --- | --- |
|  | **(mg g^-1^ protein)** | **(mg g^-1^ protein)** | **(mg g^-1^ protein)** | **(mg g^-1^ protein)** | **(mg g^-1^ protein)** |
| WT | 14.9 ± 3.2 | 1440 ± 190 | 3.9 ± 3.2 | 0.48 ± 0.11 | 0.86 ± 0.43 |
| Δ*mpfA* | 19.3 ± 2.1 | 1782 ±371 | 7.8 ± 4.9 | 0.51 ± 0.14 | 0.41 ± 0.28 |
