# Supplementary material for: A Central Role for Magnesium Homeostasis during Adaptation to Osmotic Stress
Source: mBio. 2022 Feb 15;13(1):e00092-22. doi: 10.1128/mbio.00092-22 (PMC8844918; doi:10.1128/mbio.00092-22)
Supplement: TABLE S1 [file mbio.00092-22-st001.docx]

Table S1.

Strains and plasmids used in this study

| **Strain** | **Genotype** | **Reference** |
| --- | --- | --- |
| WT (CU1065) | *trpC2 attSPβ sfp^0^* | Lab stock |
| HB24928 | *ΔmpfA* | (1) |
| HB24943 | *kimA::mls* | This study |
| HB24944 | *ktrAB::neo* | This study |
| HB24946 | *ΔmpfA kimA::mls* | This study |
| HB24948 | *ΔmpfA ktrAB::neo* | This study |
| HB24950 | *ktrAB::neo kimA::mls* | This study |
| HB24953 | *ΔmpfA ktrAB::neo kimA::mls* | This study |
| HB25008 | *ΔyrkA* | This study |
| HB25010 | *ΔyhdT* | This study |
| HB25011 | *ΔyqhB* | This study |
| HB24992 | *ΔmpfA yrkA::mls* | This study |
| HB24994 | *ΔmpfA yhdT::mls* | This study |
| HB24993 | *ΔmpfA yqhB::mls* | This study |
| HB24990 | *ΔmpfA amyE::PxylA-mpfA* | (1) |
| HB25092 | *ΔmpfA amyE::PxylA-mgtE* | This study |
| HB24965 | *amyE::PxylA-mgtE* | (1) |
| HB26505 | *rpmH::kan* | This study |
| HB25088 | *cdaA::mls* | This study |
| HB25089 | *disA::mls* | This study |
| HB25090 | *gdpP::mls* | This study |
| HB25091 | *pgpH::mls* | This study |
| HB25095 | *pgpH::mls gdpP::kan* | This study |
| HB24958 | *lacA::Pxyl-dcas9-cam amyE::Pveg-sgRNA(mgtE)-erm* | This study |
| HB26531 | *cdaA::kan cdaS::erm disA::spec* | This study |
| **Plasmid** | **Description** | **Reference** |
| pSWEET-mpfA | Expression of *mpfA* under P_xylA_ promoter | (1) |
| pSWEET-mgtE | Expression of *mgtE* under P_xylA_ promoter | (1) |
| pDR244 | Generate markerless deletion using *cre*/lox-mediated loop-out system | Lab stock |
