# Supplementary material for: A Central Role for Magnesium Homeostasis during Adaptation to Osmotic Stress
Source: mBio. 2022 Feb 15;13(1):e00092-22. doi: 10.1128/mbio.00092-22 (PMC8844918; doi:10.1128/mbio.00092-22)
Supplement: TABLE S2 [file mbio.00092-22-st002.docx]

Table S2.

Primer oligonucleotides

| **Number** | **Name** | **Sequence** |
| --- | --- | --- |
| **Primers used for strain verification** | | |
| 8776 | yhdP _F | ATAACGTGATGGTTGGGAG |
| 8777 | yhdP_R | TCAAATACACTACATGGCG |
| 8900 | ktrAB_check_F | CGATTGGCTGTTTTATTAACA |
| 8901 | ktrAB_check_R | TAATGCTGATAATCTGGAACT |
| 8902 | kimA_check_F | CGTGAGAGGAGATGAATGAA |
| 8903 | kimA_check_R | TTCCAATATCTCCCGCAC |
| 8778 | yqhB_F | ATTAGTTCGCCGAGAAGAATTA |
| 8779 | yqhB_R | ACGGAAGAATATCACCAGGTG |
| 8780 | yhdT_F | ATGAAGTTGCATTTTGCGTG |
| 8781 | yhdT_R | TGGCTCTTTTATATGTGGCA |
| 8784 | yrkA_F | ACACCAGAAAATTCAGGTG |
| 8785 | yrkA_R | TGTCGGGCTTTACTATGAAG |
| 9235 | cdaA_F | ACTGTCAAAAACGGCAAG |
| 9236 | cdaA_R | TTCAAAGTTTTTGGTCTGCCG |
| 9237 | disA_F | TTGAAATTCAGGCACTCATCT |
| 9238 | disA_R | CACCCAGTTCACAACGT |
| 9239 | gdpP_F | TGCCTTTAGAAAAAGGCCAAA |
| 9240 | gdpP_R | CTGAACCAAATAAACGGCCG |
| 9241 | pgpH_F | CTTGATGAAGCACAGAATACCAC |
| 9242 | pgpH_R | ATGACATCTGTCGGAGCA |
| 9235 | rpmH_F | AGAAATAAAAGCACTAGTGAAGT |
| 9236 | rpmH_R | TATGAACATGAGATGCATCTG |
| 9446 | cdaS_F | TCTTTCATACATTGATCTCCGTTTC |
| 9447 | cdaS_R | ACGGTACTATAAATGCATAAACAGG |
| **Primers used for real-time qPCR** | | |
| 8726 | gyrA-RT-F | GGCGGCCATGCGTTATACAG |
| 8727 | gyrA-RT-R | GCCATACCTACCGCAATGCC |
| 9185 | mgtE-qPCR-F | CTGCGAGACGGGAAAATCAGG |
| 9186 | mgtE-qPCR--R | CAGCTCCCCGATCATATCAGTG |
